# Fear of COVID-19 and its association with mental health-related factors: systematic review and meta-analysis

**DOI:** 10.1192/bjo.2022.26

**Published:** 2022-03-21

**Authors:** Zainab Alimoradi, Maurice M. Ohayon, Mark D. Griffiths, Chung-Ying Lin, Amir H. Pakpour

**Affiliations:** Social Determinants of Health Research Center, Research Institute for Prevention of Non-Communicable Diseases, Qazvin University of Medical Sciences, Iran; Stanford Sleep Epidemiology Research Center (SSERC), School of Medicine, Stanford University, California, USA; International Gaming Research Unit, Department of Psychology, Nottingham Trent University, UK; Institute of Allied Health Sciences, College of Medicine, National Cheng Kung University, Taiwan; Biostatistics Consulting Center, National Cheng Kung University Hospital, College of Medicine, National Cheng Kung University, Taiwan; Department of Public Health, College of Medicine, National Cheng Kung University, Taiwan; and Department of Occupational Therapy, College of Medicine, National Cheng Kung University, Taiwan; Social Determinants of Health Research Center, Research Institute for Prevention of Non-Communicable Diseases, Qazvin University of Medical Sciences, Iran; and Department of Nursing, School of Health and Welfare, Jönköping University, Sweden

**Keywords:** COVID-19, fear, anxiety disorders, depressive disorders, sleep disorders

## Abstract

**Background:**

The severity of COVID-19 remains high worldwide. Therefore, millions of individuals are likely to suffer from fear of COVID-19 and related mental health factors.

**Aims:**

The present systematic review and meta-analysis aimed to synthesize empirical evidence to understand fear of COVID-19 and its associations with mental health-related problems during this pandemic period.

**Method:**

Relevant studies were searched for on five databases (Scopus, ProQuest, EMBASE, PubMed Central, and ISI Web of Knowledge), using relevant terms (COVID-19-related fear, anxiety, depression, mental health-related factors, mental well-being and sleep problems). All studies were included for analyses irrespective of their methodological quality, and the impact of quality on pooled effect size was examined by subgroup analysis.

**Results:**

The meta-analysis pooled data from 91 studies comprising 88 320 participants (mean age 38.88 years; 60.66% females) from 36 countries. The pooled estimated mean of fear of COVID-19 was 13.11 (out of 35), using the Fear of COVID-19 Scale. The associations between fear of COVID-19 and mental health-related factors were mostly moderate (Fisher's *z* = 0.56 for mental health-related factors; 0.54 for anxiety; 0.42 for stress; 0.40 for depression; 0.29 for sleep problems and –0.24 for mental well-being). Methodological quality did not affect these associations.

**Conclusions:**

Fear of COVID-19 has associations with various mental health-related factors. Therefore, programmes for reducing fear of COVID-19 and improving mental health are needed.

## COVID-19 pandemic and mental health

The entire world has experienced the threat of COVID-19 since the initial outbreak in China at the end of 2019. The World Health Organization^1^ announced COVID-19 as a global pandemic in March 2020, and the COVID-19 infection rate still remains high globally because of its several mutations.^2,3^ Indeed, at the time of writing (August 2021), the number of confirmed COVID-19 cases was near to 0.2 billion and the number of deaths had exceeded 4 million across 220 countries and territories worldwide.^4^ To control COVID-19 infection in an efficient and timely manner, different techniques have been used to rapidly develop COVID-19 vaccines.^5^ Unfortunately, empirical evidence shows that implementing COVID-19 vaccination programmes is not without difficulties, including the low willingness by some individuals in relation to vaccine uptake.^6–9^ Moreover, the speed that COVID-19 mutates into different variants is high,^3^ which may restrict the efficiency of the current COVID-19 vaccines in controlling the infection rate. Therefore, the uncontrolled pandemic causes several severe problems for individuals globally, and one of these problems relates to mental health.

Because the global reach of the COVID-19 pandemic is unprecedented, with many different and vigorous infection control methods (e.g. lockdown) implemented,^10–12^ mental health problems (e.g. psychological distress) during the COVID-19 pandemic have been high.^13–17^ Moreover, one of the primary triggers for mental health problems during this period is fear of COVID-19.^18^ More specifically, COVID-19 is a new type of infection, and different stakeholders (including governments, healthcare providers, policy makers and scientists) require information and data to help fight the consequences of the disease. Therefore, fear is likely to develop among many individuals because of the life-threatening effects of COVID-19 and the fact that the many methods implemented to control the infection rate have had varied levels of success. Given that the COVID-19 infection and its severity are unlikely to be under control in the short term,^19,20^ it is important to accumulate scientific evidence regarding fear of COVID-19 and its association with mental health-related factors. Using the empirical data regarding the associations between fear of COVID-19 and mental health-related factors, healthcare providers and policy makers can understand the importance of controlling fear of COVID-19 during the pandemic period, and implement initiatives to prevent potential mental health problems.

## Factors included in the present systematic review and meta-analysis

In the present systematic review and meta-analysis, mental health-related factors, including depression, anxiety, stress, sleep problems, mental health-related factors and mental well-being, were identified, analysed and discussed. These factors were included because they are important factors that affect an individual's ability to live a happy and healthy life. For example, depression, anxiety, stress and mental health-related factors have been found to be important factors that jeopardise sleep quality and physical health.^21–23^ Moreover, sleep has been identified as an important and essential daily activity for individuals to maintain daily functions.^24^ In this regard, when individuals encounter any problem related to one of these mental health-related factors, their quality of life and well-being is jeopardised, and a minority of individuals may develop serious health problems.^25–27^

More specifically, when individuals encounter a mental health-related problem, they need additional support from community and/or healthcare systems to assist them in coping with both mental and physical health problems. Moreover, individuals with mental health-related problems may have decreased productivity, resulting in fewer contributions to society.^25–27^ As a result, society and healthcare system have higher levels of burden if the society and community have larger proportion of residents living with mental health-related problems.^25–27^ Therefore, understanding the associations between fear of COVID-19 and the aforementioned mental health-related factors are of great importance during the COVID-19 pandemic period.

## Purpose and aim of the present systematic review and meta-analysis

Consequently, the present systematic review and meta-analysis was carried out to provide empirical evidence for healthcare workers and related stakeholders (e.g. government authorities, policy makers) to better understand fear of COVID-19 and its associations with mental health-related problems during the pandemic period. The main aims of the review were to (a) estimate the mean fear of COVID-19 scores in the context of the COVID-19 pandemic from studies, using the Fear of COVID-19 Scale (FCV-19S); (b) assess the association of fear of COVID-19 with mental health-related factors (including depression, anxiety, stress, sleep problems, mental health-related factors and mental well-being) in the context of the COVID-19 pandemic; (c) identify potential sources of heterogeneity and its possible sources for the aforementioned mean and association estimations; and (d) identify moderators in the mean estimation and association between fear of COVID-19 and mental health-related factors.

## Method

### Design and protocol registration

The project was registered in the International Prospective Register of Systematic Reviews (PROSPERO) website (registration number CRD42020188890.^28^ The study's findings are reported according to the Preferred Reporting Items for Systematic Reviews and Meta-Analyses (PRISMA) guidelines.^29^

### Search strategy

From December 2019 to June 2021, five academic databases (i.e. Scopus, ProQuest, EMBASE, PubMed Central and ISI Web of Knowledge) were systematically searched. COVID-19-related fear, in combination with mental health-related keywords including anxiety, depression, psychological distress, mental well-being and sleep problems, were used to develop search syntax. The relevant search terms were extracted from PubMed Medical Subject Headings and published studies. Search syntax was customised for the aforementioned academic databases based on their search attributes. Additionally, hand searches were performed by reading reference lists of included studies and published systematic reviews to increase the retrieval of relevant studies.

### Outcomes

The main outcomes of the present systematic review were mean of fear of COVID-19 was estimated in the context of the COVID-19 pandemic based on FCV-19S scores internationally; and the association of fear of COVID-19 with other mental health-related factors (mentioned below), which was calculated in the context of the COVID-19 pandemic. Moreover, fear of COVID-19 was defined as the threatening stimulus of COVID-19 resulting in the triggering of unpleasant emotional state among individuals.^30^

The secondary outcomes were to identify potential sources of heterogeneity and its possible sources, moderators in mean estimation fear of COVID-19, and moderators in the association of fear of COVID-19 with other mental health-related factors. The other mental health-related factors were defined as follows: depression, defined as lacking interests of engaging in activities and having low mood without pleasure;^31^ anxiety, defined as having excessive worry on various activities, events, topics and daily errand;^31^ stress, defined as a nonspecific response from an individual's body that reacts to any demands;^32^ sleep problems, defined as sleep disorders in a broad category with some subcategories, including intrinsic, extrinsic and disturbances of circadian rhythm;^33^ mental health-related factors, defined as perceived discomfort from response to stressors that is hard to cope with;^34^ and mental well-being, defined as the psychological processes of individuals that promote life outcomes in a positive way, including happiness and growth toward optimal development.^35^

### Eligibility criteria

All peer-reviewed observational studies published in the English language were considered eligible if relevant data regarding mean scores regarding fear of COVID-19 (on the FCV-19S) and their association with mental health problems and/or distress (e.g. anxiety, depression, mental health-related factors, mental well-being and sleep problems) were reported. To be included, the fear of COVID-19 and mental health-related factors had to have been assessed by valid and reliable psychometric scales. No limitation was exerted regarding participants’ characteristics. More specifically, studies were excluded if they had other study designs (intervention studies, letters to the editor, editorials, qualitative studies, systematic reviews), did not report numerical findings regarding the selected outcome measures, did not have valid or reliable measures for assessing the selected variables and were non-English language publications.

### Screening process and study selection

First, titles and abstracts of all retrieved papers were independently screened based on eligibility criteria, by two of the research team. Then full texts of potentially eligible papers were downloaded and reviewed for final selection. During this process, relevant studies were selected. This stage was carried out independently by two members of the research team. The kappa score showed strong agreement between these reviewers (*κ* = 0.83).

### Quality assessment

The methodological quality of the included papers was assessed with the Newcastle–Ottawa Scale (NOS) checklist.^36^ The NOS checklist assesses the methodological quality of papers in three domains of selection, and comparability with seven items for cross-sectional studies. Studies yielding fewer than five points are classified as having a high risk of bias.^36^ No studies in the present review were excluded on the basis of poor methodological quality. However, the impact of quality on pooled effect size was assessed by subgroup analysis. Quality assessment of included studies were carried out independently by two members of the research team. The kappa score showed strong agreement between these reviewers (*κ* = 0.78).

### Data extraction

A predefined Microsoft Excel version 2016 for Windows spreadsheet was designed to extract data based on the study aims and selected outcomes. Data extracted included the first author's name, publication date, title of the study, country of research, target population of study (categorised as general population, healthcare professionals and patients with COVID-19), sample size, study design, fear of COVID-19 measures and scores (including mean and s.d.), mental health-related factor outcomes measures and their association with fear of COVID-19, and NOS score (i.e. methodological quality). Data extraction of included studies were carried out independently by two members of the research team. The kappa score showed strong agreement between these reviewers (*κ* = 0.75).

It should also be noted that study selection, quality assessment and data extraction were processes performed independently by two reviewers. Disagreements regarding whether a study should be included or not, methodological quality assessment of included studies and data extraction were resolved through discussion by independent reviewers.

### Data synthesis

A quantitative synthesis using Stata software version 14 for Windows was conducted. Meta-analysis was run with random effect model because the included studies were taken from different populations, and both within-study and between-study variances should be accounted for.^37^ The *Q* Cochrane statistic was used to assess heterogeneity. Also, the severity of heterogeneity was estimated with the *I*^2^ index. Heterogeneity is interpreted as mild when *I*^2^ is <25%, moderate when *I*^2^ is 25–50%, severe when *I*^2^ is 51–75% and highly severe when *I*^2^ is >75%.^38^ Two key measures were selected for present study:

- Mean score of fear of COVID-19 (using the FCV-19S): The numerical findings regarding means and standard deviations of fear of COVID-19 scores were reported consistently in 71 included studies. This key measure and its 95% confidence interval were reported.
- Correlation of fear of COVID-19 with other mental health-related factors: Other mental health-related factors were defined as depression, anxiety, stress, sleep problems, mental health-related factors and mental well-being. Pearson's correlation coefficient was the selected effect size for meta-analysis in assessing the associations between fear of COVID-19 and these mental health-related factors. Because of the potential instability of variance, Pearson's *r* correlation coefficient was converted to Fisher's *z*-statistic. Consequently, all analyses were performed with Fisher's *z*-values as effect sizes.^39,40^ Fisher's *z*-transformation was applied by using the following formula: *z* = 0.5 × ln[(1 + *r*) − (1 − *r*)]. The s.e. of *z* was calculated based on the following formula: s.e. *z* = 1/√(*n* − 3).^41^ Therefore, the selected measure of effect (selected for current meta-analysis) is expressed as Fisher's *z*-score and its 95% confidence interval. Moreover, Fisher's *z* at 0.1 is defined as weak, 0.11–0.3 is defined as weak to moderate, 0.3 is defined as moderate, 0.31–0.49 is defined as moderate to strong and ≥0.5 is defined as strong. For assessing moderator analysis, subgroup analysis or meta-regression was carried out. Funnel plot and the Begg's test were used to assess publication bias.^42^ The jackknife method was used for sensitivity analysis^43^ and to determine the effect of individual studies on the outcome. The jackknife method is also known as the ‘one-out method’, and was used to evaluate the quality and consistency of the results. More specifically, significant changes can be evaluated by removing each study individually.^44^

## Results

### Screening and selection process

**Fig. 1:**
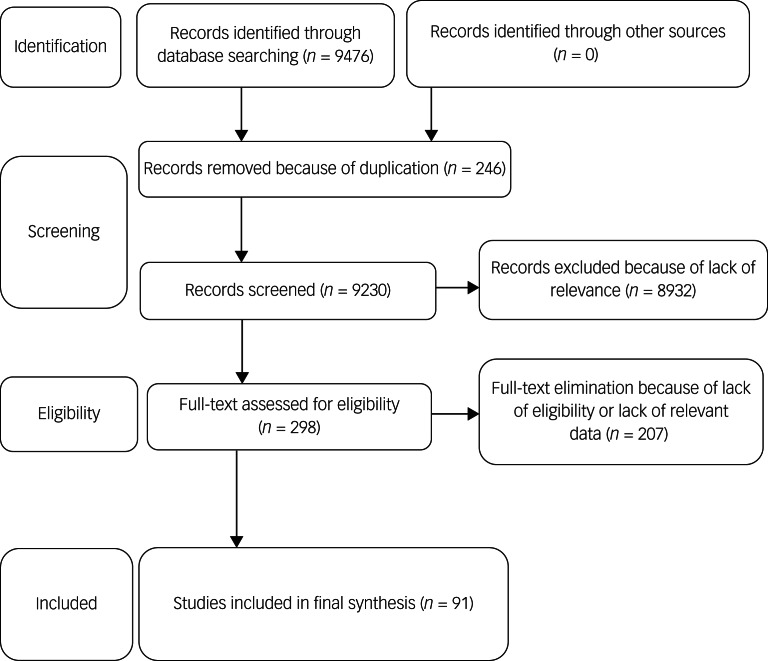
Preferred Reporting Items for Systematic Reviews and Meta-Analyses flow chart of selected studies.

The initial search of five databases identified 9476 papers: Scopus (*n* = 1768), Web of Science (*n* = 1200), PubMed (*n* = 1240), EMBASE (*n* = 5012) and ProQuest (*n* = 256). After removing 246 duplicates, 9230 papers were screened based on the title and abstract. Finally, 298 papers deemed as eligible had their full texts were reviewed. During this process, 91 papers met the eligibility criteria and were pooled in the meta-analysis. Fig. 1 shows the search process based on the PRISMA (2009) flow chart.

### Study description

A total of 91 studies were included in the final analysis. Included studies comprised 88 320 participants from 36 countries (Australia, Bangladesh, Brazil, Canada, China, Ecuador, Egypt, Germany, Greece, India, Iran, Israel, Italy, Japan, Jordan, Korea, Lebanon, Malaysia, Mexico, The Netherlands, Pakistan, Paraguay, Peru, the Philippines, Poland, Romania, Russia, Taiwan, Turkey, Saudi Arabia, Singapore, Spain, United Arab Emirates, UK, USA and Vietnam). Turkey (*n* = 10 papers), Iran (*n* = 6 papers), Bangladesh (*n* = 5 papers) and Pakistan (*n* = 5 papers) had the highest number of studies. Almost all studies (*n* = 90) employed a cross-sectional design. Seven papers collected data during national lockdown periods in their respective countries. The target populations in the studies were either the general population (*n* = 80) or healthcare professionals (*n* = 11). Sample size varied between 58 and 10 067 participants. Mean age of participants was 38.88 years. Approximately 61% of the total number of participants were females. The FCV-19S, developed by Ahorsu et al,^45^ was the most frequently used instrument to assess COVID-19-related fear in 71 studies. Mental health-related factors assessed included sleep problems (*n* = 9), depression (*n* = 49), anxiety (*n* = 48), stress (*n* = 19), psychological distress (*n* = 6) and mental well-being (*n* = 3). Different valid and reliable psychometric instruments were used to assess these outcomes. Table 1 provides the summary characteristics of all included studies.

**Table 1:** Summary characteristics of included studies

Study	Collection date	Country	Design	Participant group	Lockdown period	Sample size	Gender group	Female, %	Age, years	Fear of COVID-19 Scale	Psychological measures
^46^		India	Cross-sectional	General population	Yes	625	Both	37.80	17–23	FCV-19S	PSS-4; WHO-5
^47^		Bangladesh	Cross-sectional	General population		262 male and 259 female	Both	49.71	24.78	FCV-19S	PSQI; PSS-10
^48^	15 June to 15 July 2020	Saudi Arabia	Cross-sectional	General population	Yes	1030	Both	76.10	36.40	FCV-19S	HADS
^49^	18 March to 15 May 2020	Spain	Cross-sectional	General population		124	Both	48.40	41.20	FCV-19S	STAI
^50^		Iran	Cross-sectional	Treatment-seeking patients with principal diagnoses of anxiety disorders		300	Both	58.70	36.12	COVID-19 Phobia Scale	PHQ-4; SHAI
^51^	May 2020 to June 2020	Pakistan	Cross-sectional	Older population		310	Both	31.90	50−80	FCV-19S	HADS
^52^	April and May 2020	Turkey	Cross-sectional	Undergraduate and graduate university students		506	Both	78.70	21.69	FCV-19S	DASS
^53^		China	Longitudinal	College students		867	Both	69.00	20.17	Fear of contagion	PSS-10
^54^	June 2020	Australia	Cross-sectional	General population		516	Both	62	41.10	FCV-19S	Kessler Psychological Distress Scale
^55^		Pakistan	Time-lagged	General population		267	Both	34.00		FCV-19S	PHQ-9
^56^	29 June to 9 August 2020	Germany	Cross-sectional	General population		515	Both	90.30		FCV-19S	SHAI
^57^	11 April to 11 May 2020	Saudi Arabia	Cross-sectional	General population		1029	Both	47.30	33.70	FCV-19S	HADS
^58^	11 and 20 April 2020	Saudi Arabia	Cross-sectional	General population		693	Both	42	34.75	FCV-19S	HADS
^59^		Ecuador	Cross-sectional	Undergraduate students	Yes	640	Both	72.00	21.69		DASS
^60^	31 January to 9 February 2020	China	Cross-sectional	General population		3233	Both	54.38	31.71	FCV-19S	Psychological questionnaire for emergent events of public health
^61^	1 April to 30 April 2020	Iran	Cross-sectional	General population		413	Both	38.00	57.72	FCV-19S	ISI; PHQ-9
^62^	11 and 15 May 2020	Poland	Cross-sectional	Cancer patients		306	Both	54.58	63.00	FCV-19S	Numeric Anxiety Scale
^63^	April and May 2020	Iran	Cross-sectional	General population		651	Both	62.40		FCV-19S	Anxiety Sensitivity Questionnaire
^64^		Philippines	Cross-sectional	Front-line nurses		261	Both	73.56	30.95	FCV-19S	
^65^	March and April 2020	Iran	Cross-sectional	Pregnant women		222	Female	100.00	29.10	FCV-19S	DASS
^66^	31 March to 21 April 2020	Hong Kong	Cross-sectional	General population		219	Both	74.90	23.17	COVID-19 Fear (Higher Education) Scale	GAD-7
^67^	19 June and 10 July 2020	Brazil	Cross-sectional	Pregnant women		204	Female	100.00	30.12	FCV-19S	PDSS-24; PSS-10
^68^		Japan	Cross-sectional	General population		450	Both	35.00	48.13	FCV-19S	HADS
^69^	1 and 30 June 2020	Australia	Cross-sectional	General population		58	Both	61.80	41.30	FCV-19S	
^70^	20 September and 30 October	Vietnam	Cross-sectional	General population		1510	Both	56.70	>18	Fear and anxiety of COVID-19	PROMIS six-item Sleep Disturbance Scale; Kessler Psychological Distress Scale
^71^		Japan	Cross-sectional	General population		222	Both	43.70	>18	FCV-19S	DASS
^72^		The Netherlands	Cross-sectional	General population		546	Both	44.69	>18	Fear of the Coronavirus Questionnaire	DASS
^73^	June and November 2020	Korea	Cross-sectional	General population		203	Both	57.64	39.63	FCV-19S	HADS
^74^		Singapore	Cross-sectional	General population		413	Both	65.40	69.09	COVID-19 Fear Inventory	GDS-15; GAI-SF
^75^	1–25 May 2020	UK	Cross-sectional	General population	Yes	165	Both	61.00	15.90	Coronavirus Inventory	HADS; PSS
^76^	16–23 August 2020	Jordan	Cross-sectional	Healthcare workers		365	Both	55.60	>20	FCV-19S	DASS
^77^	8 October to 26 November 2020	Turkey	Cross-sectional	General population		3287	Both	56.70	>16	FCV-19S	DASS
^78^	4–25 August 2020	Japan	Cross-sectional	General population		6750	Both	63.50	>18	FCV-19S	GAD-7; Kessler Psychological Distress Scale
^79^	15 March and 30 April 2020	Turkey	Cross-sectional	General population		431	Both	66.60	33.81	FCV-19S	
^80^	9–13 July 2020	India	Cross-sectional	General population		163	Both		26.64	FCV-19S	CESD; GAD-7
^81^	April to June 2020	Lebanon	Cross-sectional	Individuals with physical disabilities		118	Both	11.90	37.75	FCV-19S	Hopkins Symptom Checklist 25
^82^	May 2020	UK	Cross-sectional	General population		226	Both		29.80	FCV-19S	
^83^	19–21 March 2020	Paraguay	Cross-sectional	General population		1077	Both	68.71	30.95	FCV-19S	HADS
^84^		Saudi Arabia	Cross-sectional	General population		255	Both	88.00	32.96	FCV-19S	DASS
^85^	15 April and 15 May 2020	Turkey	Cross-sectional	General population		362	Both	66.90	26.89	FCV-19S	HADS
^86^	20–31 May 2020	Turkey	Cross-sectional	General population		355	Both	71.50	22.41	FCV-19S	SCL-90
^87^	May 2020	China	Cross-sectional	General population		1794	Both	43.80	15.26	FCV-19S	Youth Self-Rating Insomnia Scales
^88^	27 April and 10 May 2020	Romania	Cross-sectional	General population		809	Both	65.40	32.61	FCV-19S	Short Depression-Happiness Scale; PSS
^89^	July to October 2020	USA	Cross-sectional	Patients with ovarian cancer		100	Female	100.00	55.03	FCV-19S	DASS
^90^	25 May to 12 June 2020	Malaysia	Cross-sectional	General population		255	Both	65.50		FCV-19S	DASS
^91^	1–10 April 2020	Bangladesh	Cross-sectional	General population		10067	Both	43.90	>10	FCV-19S	ISI
^92^	17 September and 10 November 2020	Canada	Cross-sectional	Ophthalmology tertiary care centre		160	Both	69.40		FCV-19S	
^93^	30 June to 29 September 2020	Egypt	Cross-sectional	Patients with diabetes mellitus		200	Both	63.00	48.40	FCV-19S	
^94^	1 April to 30 May 2020	Canada	Cross-sectional	General population		434	Male	0.00	39.76	FCV-19S	
^95^	2 and 24 July 2020	UK	Cross-sectional	People with chronic pain		555	Both	86.30	40.00	FCV-19S	PHQ-9
^96^ Study 1	Apr 2020	Pakistan	Cross-sectional	General population		316	Both	71.00		Fear of COVID-19	Cole Insomnia Scale
^96^ Study 2	May 2020	Pakistan	Cross-sectional	General population		421	Both	74.00		Fear of COVID-19	Cole Insomnia Scale
^97^	10 May to 9 June 2020	Egypt	Cross-sectional	Physicians		320	Both	63.40	34.60	FCV-19S	HADS
^98^		Italy	Cross-sectional	General population		1200	Both	76.60	39.59	FCV-19S	SCL-90
^99^		Turkey	Cross-sectional	Healthcare providers		208	Both	27.90	29.00	FCV-19S	
^100^	1–30 May 2020	United Arab Emirates	Cross-sectional	General population		433	Both	35.8	21	FCV-19S	Kessler Psychological Distress Scale
^101^	13–22 February 2020	China	Cross-sectional	General population		4164	Both	48		COVID-19 Fear Screening Scale	PHQ-9
^102^		Greece	Cross-sectional	General population		103	Both	61.17	>60	FCV-19S	
^103^	10–13 April 2020	Greece	Cross-sectional	General population	Yes	3029	Both	71.9	>18	FCV-19S	PHQ-9; GAD-7
^104^	17 April to 3 May 2020	Italy	Cross-sectional	Dentists		735	Both	32.7	44.8	FCV-19S	DSM-5 Severity Measure for Depression–Adult
^45^		Iran	Cross-sectional	General population		717	Both	42	31.25	FCV-19S	HADS
^105^	18–21 March 2020	Italy	Cross-sectional	General population		249	Both	92	34.5	FCV-19S	HADS
^106^	23-30 April 2020	Bangladesh	Cross-sectional	General population		232	Both	45.3	18-25	FCV-19S	DASS
^107^	March to April 2020	Turkey	Cross-sectional	General population		960	Both	69.1	29.74	FCV-19S	DASS
^108^	17–23 April 2020	Peru	Cross-sectional	General population		546 Females and 28 males	Female	65.63	38.37	FCV-19S	PHQ-9; GAD-7
^109^		Malaysia	Cross-sectional	General population		228	Both	71.1	26	FCV-19S	DASS
^110^		Russia	Cross-sectional	General population		939	Both	80.8	21.8	FCV-19S	
^111^		Pakistan	Cross-sectional	Nurses		380	Both	84.21	31.5	FCV-19S	Cavanagh Psychological Distress Scale
^112^		Mexico	Cross-sectional	Hospital staff		2860	Both	57.4	35.4	FCV-19S	
^113^	1 April to 30 May 2020	Bangladesh	Cross-sectional	Front-line doctors		370	Both	39.7	30.5	FCV-19S	
^114^	May 2020	Greece	Cross-sectional	General population	Yes	538	Both	77.9	43.05	FCV-19S	GAD-7
^115^	7 March and 21 April 2020	Iran	Cross-sectional	Pregnant women		290 Female and 290 male	Female	50	29.24	FCV-19S	HADS
^116^	15 May 2020	Japan	Cross-sectional	General population		629	Both	49.13	12.96	FCV-19S	PHQ-9; GAD-7
^117^	10–23 May 2020	Pakistan	Cross-sectional	General population	Yes	501	Both	41.5	>25	FCV-19S	
^118^		India	Cross-sectional	General population		600	Both	61	38.76	FCV-19S	Warwick–Edinburgh Mental Well-Being Scale
^119^	June to July 2020	Turkey	Cross-sectional	Nursing students		234	Both	67.9	20.12	FCV-19S	Beck Anxiety Inventory
^120^	March to April 2020	Israel	Cross-sectional	General population		649	Both	84.5		FCV-19S	DASS
^121^	July 2020	Spain	Cross-sectional	Healthcare workers		194	Both	83.5	45.94	FCV-19S	HADS
^122^		Russia	Cross-sectional	General population		850	Both	73.2	34.8	FCV-19S	
^123^		Poland	Cross-sectional	General population		907	Both	57.55	39.28	FOC-6	PSS
^124^	22–26 April 2020	Spain	Cross-sectional	General population		606	Both	82	21.59	FCV-19S	STAI
^125^	May to July 2020	Philippines	Cross-sectional	Nursing students		261	Both	81.2	20.7	FCV-19S	Sleep Quality Scale by Snyder
^126^		Turkey	Cross-sectional	General population		1772	Both	70	24.42	FCV-19S	Warwick–Edinburgh Mental Well-Being Scale
^127^		Israel	Cross-sectional	General population		130	Female	100	36.15	FCV-19S	Kessler Psychological Distress Scale (K10)
^128^	27 April to 5 May 2020	Italy	Cross-sectional	General population		623	Both	71.9	35.67	Multidimensional Assessment of COVID-19-Related Fears	DSM-5 Self-Rated Level 1 Cross-Cutting Symptom Measure–Adult
^129^		China	Cross-sectional	General population		907	Both	60		FCV-19S	GAD-7
^130^	1–10 April 2020	Bangladesh	Cross-sectional	General population		8550	Both	44	26.53	FCV-19S	PHQ-9
^131^		Turkey	Cross-sectional	General population		381	Both	49.4	15.36	FCV-19S	Revised Children's Anxiety and Depression Scale
^132^	23 March to 30 June 2020	Taiwan	Cross-sectional	Patients with mental illness		414	Both	44.4	46.32	FCV-19S	
^133^		Greece	Cross-sectional	General population		2970	Both	72.5	>18	FCV-19S	PHQ-9; GAD-7
^134^	27 - 28 March 2020	UK	Cross-sectional	General population		324	Both	50	34.32	FCV-19S	PROMIS-SF
^135^	15 May to 15 June 2020	Poland	Cross-sectional	General population		708	Both	57.49	33.4	FCV-19S	

FCV-19S, Fear of COVID-19 Scale; PSS, Perceived Stress Scale; WHO-5, WHO-Five Well-Being Index; PSQI, Pittsburgh Sleep Quality Index; HADS, Hospital Anxiety and Depression Scale; STAI, State-Trait Anxiety Index; PHQ, Patient Health Questionnaire; SHAI, Short Health Anxiety Inventory; DASS, Depression, Anxiety and Stress Scale; ISI, Insomnia Severity Index; GAD, Generalized Anxiety Disorder; PDSS-24, Perinatal Depression Screening Scale; PASS, Perinatal Anxiety Screening Scale; PROMIS, Patient-Reported Outcomes Measurement Information System; GDS-15, Geriatric Depression Scale; GAI-SF, Geriatric Anxiety Inventory–Short Form; CESD, Center for Epidemiologic Studies Depression Scale; SCL-90, Symptom Checklist-90; PROMIS-SF, Patient-Reported Outcomes Measurement Information System, Short Form.

### Methodological quality appraisal

**Fig. 2:**
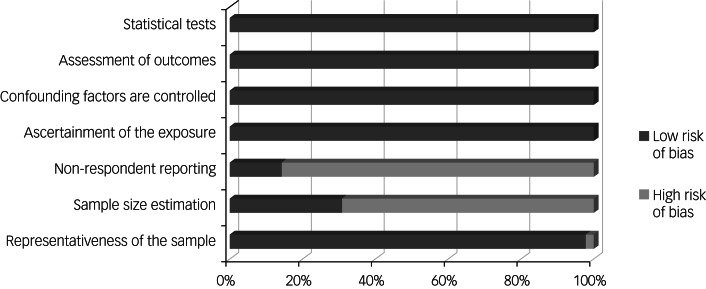
Results of quality assessment.

Methodological quality together with risk of bias were both assessed on the basis of NOS scores. The scores were then categorised as having a low risk of bias if studies acquired scores higher than 5 from maximum score of 9.^36^ Based on this criterion, all studies were categorised as being high-quality studies. The effects of study quality were further assessed and reported in subgroup analysis. The most common problems were non-representativeness of the sample owing to online sampling, not reporting sample size estimation or justification, and number of non-respondents. The results of the quality assessment are shown in Fig. 2.

### Outcome measures

#### Mean estimation of fear of COVID-19

**Fig. 3:**
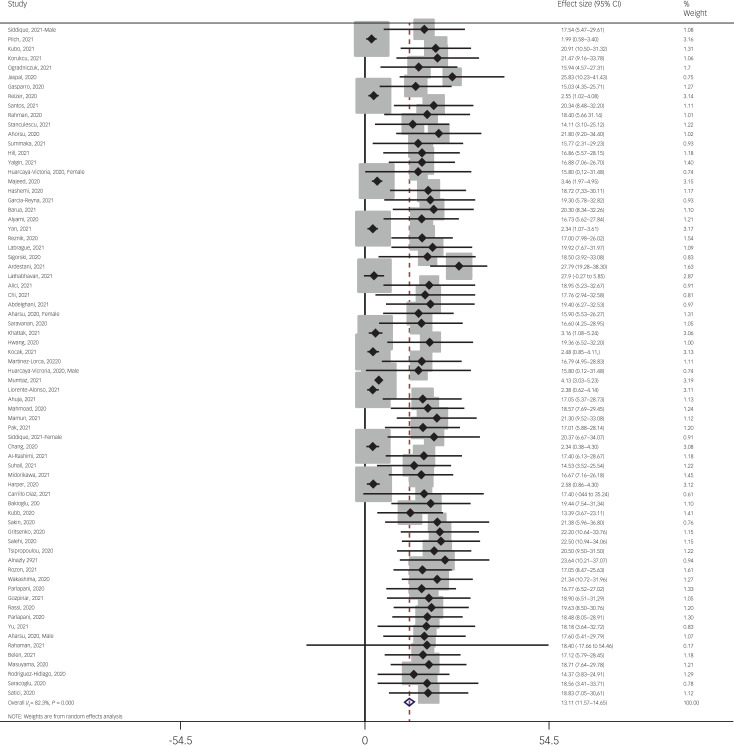
Forest plot displaying the pooled estimated mean of fear of COVID-19.

The pooled estimated mean of fear of COVID-19 was 13.11 out of 35, according to the FCV-19S (95% CI 11.57–14.65, *I*^2^ = 82.3%, *τ*^2^ = 19.02). More specifically, 76 studies reported mean fear scores, with 71 studies using the FCV-19S and five papers using other instruments. Because of the variation in the number of questions and the scoring method between the FCV-19S and the other instruments, mean estimation of fear of COVID-19 was meta-analysed using the 71 studies that utilised the FCV-19S. Fig. 3 provides the forest plot showing the pooled mean scores for fear of COVID-19.

**Fig. 4:**
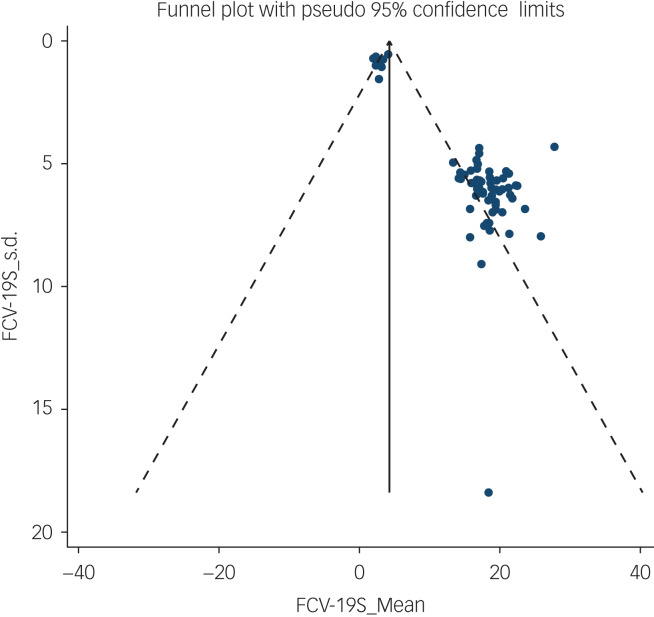
Funnel plot assessing publication bias in studies regarding pooled estimated mean of fear of COVID-19.

**Fig. 5:**
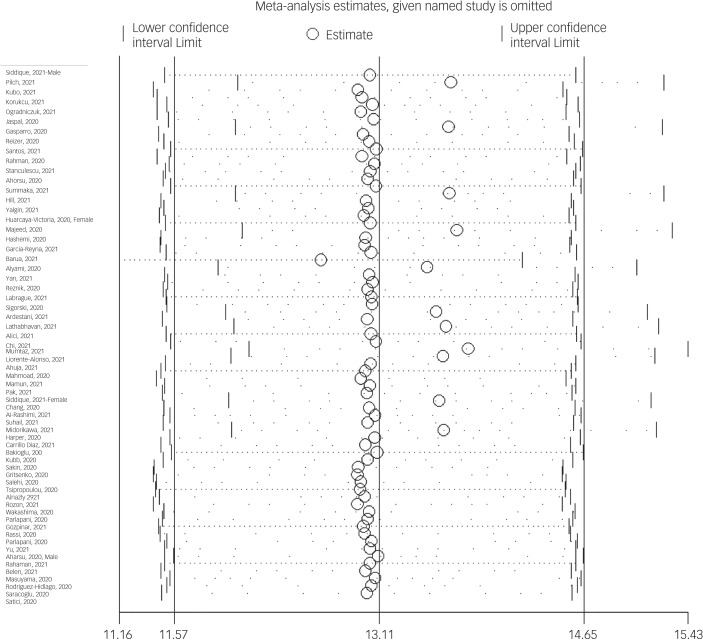
Sensitivity analysis plot assessing small study effect in pooled estimated mean of fear of COVID-19.

**Fig. 6:**
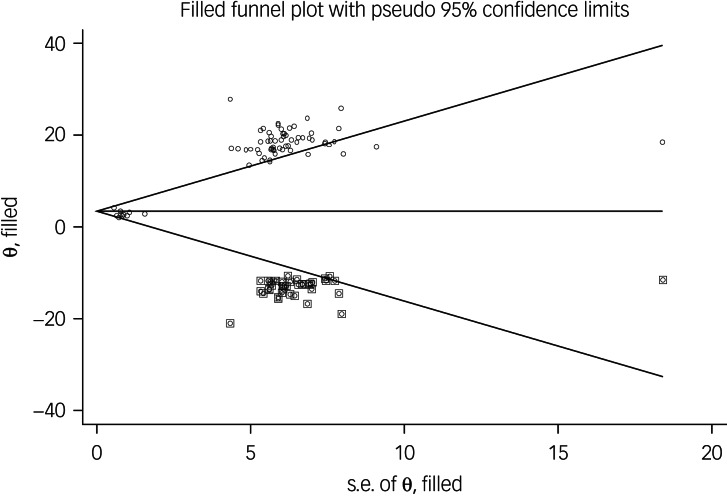
Corrected funnel plot assessing publication bias in pooled estimated mean of fear of COVID-19.

The probability of publication bias was assessed by Begg's test and funnel plot. Although the Begg's test (*P* = 0.63) did not consider publication bias, the funnel plot (Fig. 4) confirmed the probability of publication bias. Also, sensitivity analysis showed that the pooled effect size might be affected by the single-study effect (*P* < 0.001; Fig. 5). To this end, the fill-and-trim method was used to correct the results. In this method, 35 studies were imputed and the corrected results based on this method showed that pooled mean score of COVID-19-related fear was 6.20 (95% CI 4.69–7.71, *P* < 0.001). The funnel plot after trimming is shown in Fig. 6.

Subgroup analysis showed that higher mean score was observed respectively in studies with male-only participants (16.79), female-only participants (14.89) and with gender participants (13), but this difference was not significant. Other variables did not influence heterogeneity or estimated pooled mean. Results of the subgroup analysis and meta-regression are shown in Tables 2 and 3.

**Table 2:** Subgroup analysis for estimation mean for fear of COVID-19

Variable		Number of studies	Effect size (95% CI)	*I*^2^ (%)
Lockdown period	Yes	5	13.18 (4.72–21.65)	82.8
	No	66	13.19 (11.59–14.79)	79.9
Gender group	Both genders	61	13 (11.35–14.64)	82.9
	Female only	6	14.89 (5.58–24.20)	83.8
	Male only	4	16.79 (10.51–23.07)	0
Participant groups	General population	61	13.30 (11.63–14.97)	82.6
	Healthcare professionals	10	13.11 (8.64–18.13)	82.1
Overall estimated prevalence		73	13.21 (11.71–14.72)	82.4

**Table 3:** Meta-regression analysis for estimation mean for fear of COVID-19

Variable	Number of studies	Coefficient	s.e.	*P-*value	*I*^2^ residual (%)	Adjusted *R*^2^ (%)	*τ*^2^
Country	71	0.008	0.09	0.94	82.53	−1.50	45.4
Age	60	−0.11	0.11	0.33	82	1.87	41.88
Newcastle–Ottawa Scale score	71	1.46	1.20	0.65	82.49	1.41	44.1
Female % of participants	69	0.009	0.04	0.99	82.70	−1.38	47.41

#### Association between fear of COVID-19 and depression

**Fig. 7:**
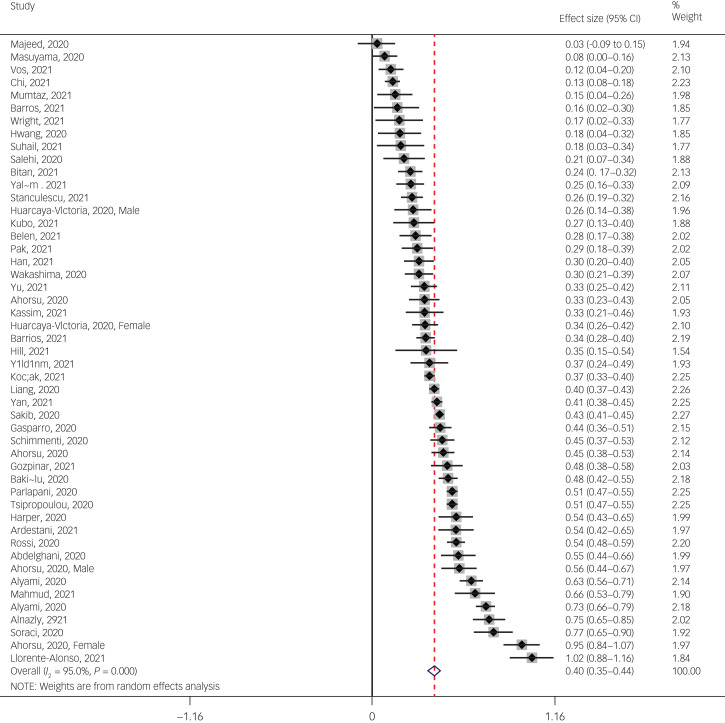
Forest plot displaying the estimated pooled Fisher's *z*-score in the association between fear of COVID-19 and depression.

**Fig. 8:**
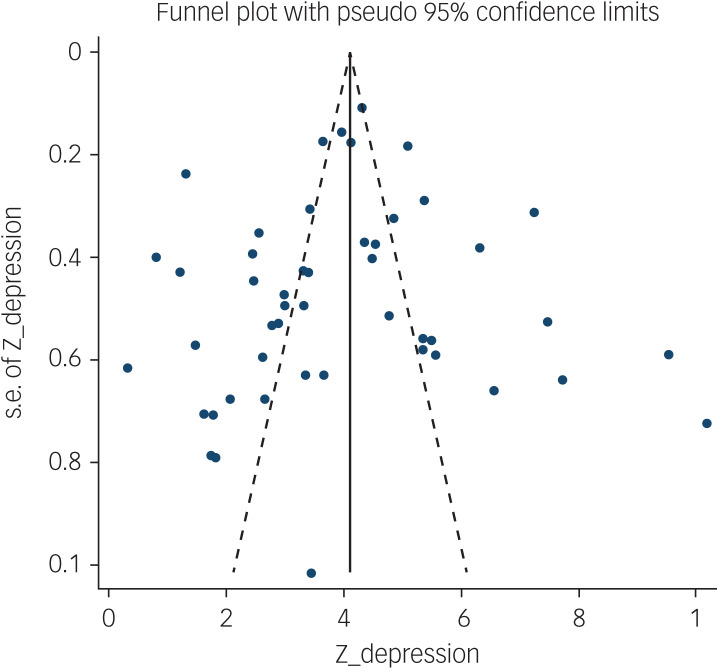
Funnel plot assessing publication bias in studies regarding the association between fear of COVID-19 and depression.

**Fig. 9:**
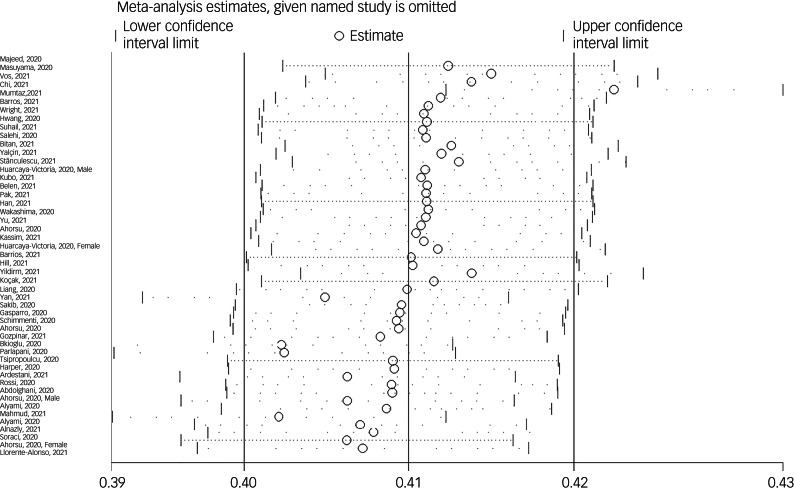
Sensitivity analysis plot assessing small study effect in the estimated pooled Fisher's *z*-score in the association between fear of COVID-19 and depression.

The association between fear of COVID-19 and depression was reported in 49 studies. The pooled estimated effect size showed moderate to strong correlation between fear of COVID-19 and depression, with a Fisher's *z*-score of 0.40 (95% CI 0.35–0.44, *I*^2^ = 95%, *τ*^2^ = 0.02). The forest plots are shown in Fig. 7. The probability of publication bias was assessed by Begg's test and funnel plot. Publication bias was not found in the association of fear of COVID-19 and depression based on Begg's test (*P* = 0.57) or funnel plot (Fig. 8). Sensitivity analysis showed that the pooled effect size was not affected by the single-study effect (*P* = 0.51; Fig. 9).

Subgroup analysis showed that association between fear of COVID-19 and depression was significantly higher among healthcare professionals compared with the general population (0.68 *v*. 0.37). Also, a higher association was observed among studies with male-only participants (0.61) compared with studies with female-only participants (0.32) and both gender participants (0.40), but this difference was not significant. Other variables did not influence heterogeneity or estimated pooled Fisher's *z*-score. Results of the subgroup analysis and meta-regression are shown in Tables 4 and 5.

**Table 4:** Subgroup analysis for association between fear of COVID-19 and mental health-related factor outcomes

		Depression			Anxiety			Stress			Sleep problems			Mental health-related factors		
Variable		Number of studies	Effect size (95% CI)	*I*^2^ (%)	Number of studies	Effect size (95% CI)	*I*^2^ (%)	Number of studies	Effect size (95% CI)	*I*^2^ (%)	Number of studies	Effect size (95% CI)	*I*^2^ (%)	Number of studies	Effect size (95% CI)	*I*^2^ (%)
Lockdown period	No	47	0.40 (0.35–0.44)	95	44	0.53 (0.46–0.60)	97.2	16	0.44 (0.36–0.52)	93.5	9	0.29 (0.22–0.37)	92.4	6	0.56 (0.34–0.77)	98.5
	Yes	2	0.35 (0.02–0.68)	94.3	4	0.70 (0.50–0.91)	96.5	3	0.33 (0.26–0.39)	35.2						
Gender Group	Both genders	42	0.40 (0.35–0.44)	95.1	42	0.55 (0.48–0.63)	97.8	18	0.42 (0.35–0.49)	92.9	9	0.29 (0.22–0.37)	92.4	5	0.57 (0.33–0.80)	98.8
	Female only	5	0.32 (0.19–0.46)	83.1	4	0.52 (0.40–0.65)	75.5	1	0.62 (0.42–0.82)	−				1	0.51 (0.34–0.68)	−
	Male only	2	0.61 (−0.07 to 1)	98.5	2	0.41 (0.33–0.49)	0									
Measure of fear	Fear of COVID-19 Scale	43	0.41 (0.36– 0.45)	95.3	42	0.55 (0.48– 0.63)	97.7	15	0.47 (0.40– 0.54)	90.2	5	0.28 (0.15– 0.40)	95.1	5	0.62 (0.33– 0.90)	98.5
	Other	6	0.33 (0.22– 0.44)	91.2	6	0.48 (0.29– 0.66)	94.7	4	0.27 (0.14– 0.40)	89.3	4	0.32 (0.22– 0.41)	81.8	1	0.27 (0.22– 0.32)	−
Participant groups	General population	45	0.37 (0.33– 0.42)	94.8	44	0.53 (0.46– 0.60)	97.7	18	0.41 (0.34– 0.47)	91.3	9	0.29 (0.22– 0.37)	92.4	5	0.41 (0.30– 0.52)	93.4
	Healthcare professionals	4	0.68 (0.45– 0.92)	95.2	4	0.67 (0.35– 0.99)	96.5	1	0.76 (0.66– 0.86)	−				1	1.28 (1.17– 1.38)	−
	Overall estimated Fisher's *z*-score	49	0.40 (0.35– 0.44)	95	48	0.54 (0.48– 0.61)	97.6	19	0.42 (0.35– 0.50)	92.6	9	0.29 (0.22– 0.37)	92.4	6	0.56 (0.34– 0.77)	98.5

**Table 5:** Meta-regression analysis for moderator analysis association between fear of COVID-19 and mental health-related factor outcomes

		Number of studies	Coefficient	s.e.	*P-*value	*I*^2^ residual (%)	Adjusted *R*^2^ (%)	*τ*^2^
Depression	Country	49	−0.001	0.003	0.69	95.11	−1.99	0.04
	Age	38	0.002	0.003	0.49	96.49	−1.40	0.04
	Newcastle–Ottawa Scale score	49	0.02	0.04	0.61	95.06	−1.67	0.04
	Female % of participants	48	−0.0001	0.001	0.91	95.17	−2.31	0.04
	Measure of depression	49	−0.004	0.01	0.71	95.12	−2.04	0.04
Anxiety	Country	48	0.006	0.003	0.07	96.8	5.32	0.05
	Age	35	0.007	0.003	0.01	95.27	15.54	0.04
	Newcastle–Ottawa Scale score	48	0.09	0.05	0.05	97.32	6.53	0.05
	Female % of participants	47	0.002	0.002	0.19	97.39	1.68	0.05
	Measure of anxiety	48	−0.0001	0.009	1	97.57	−2.27	0.05
Stress	Country	17	0.001	0.004	0.71	92.99	−5.25	0.02
	Age	11	0.007	0.004	0.15	92.52	11.80	0.02
	Newcastle–Ottawa Scale score	17	0.14	0.07	0.07	91.62	15.51	0.02
	Female % of participants	17	0.0004	0.002	0.83	93	−5.87	0.02
	Measure of stress	17	0.7	0.7	0.36	92.5	−0.54	0.02
Sleep problems	Country	9	−0.0004	0.004	0.91	91.42	−15.47	0.008
	Age	5	0.004	0.002	0.11	50.18	63.58	0.002
	Newcastle–Ottawa Scale score	9	−0.06	0.06	0.34	88.16	1.30	0.007
	Female % of participants	9	0.001	0.001	0.42	93.36	−3.80	0.007
	Measure of sleep problems	9	0.009	0.02	0.61	92.15	−12.75	0.008
Mental health-related factors	Country	6	−0.01	0.02	0.57	98.70	−13.90	0.15
	Age	4	−0.01	0.03	0.73	99	−39.93	0.26
	Newcastle–Ottawa Scale score	6	−0.29	0.2	0.21	98.67	19.59	0.11
	Female % of participants	6	0.007	0.007	0.44	98.35	−5.14	0.14
	Measure of mental health-related factors	6	0.87	0.14	0.003	93.38	89.68	0.01
Mental well-being	Country	3	−0.003	0.006	0.74	−	−	−
	Lockdown period	3	0.03	0.05	0.68	−	−	−
	Female % of participants	3	−0.001	0.001	0.67	−	−	−
	Measure of mental well-being	3	−0.03	0.05	0.68	−	−	−

#### Association between fear of COVID-19 and anxiety

**Fig. 10:**
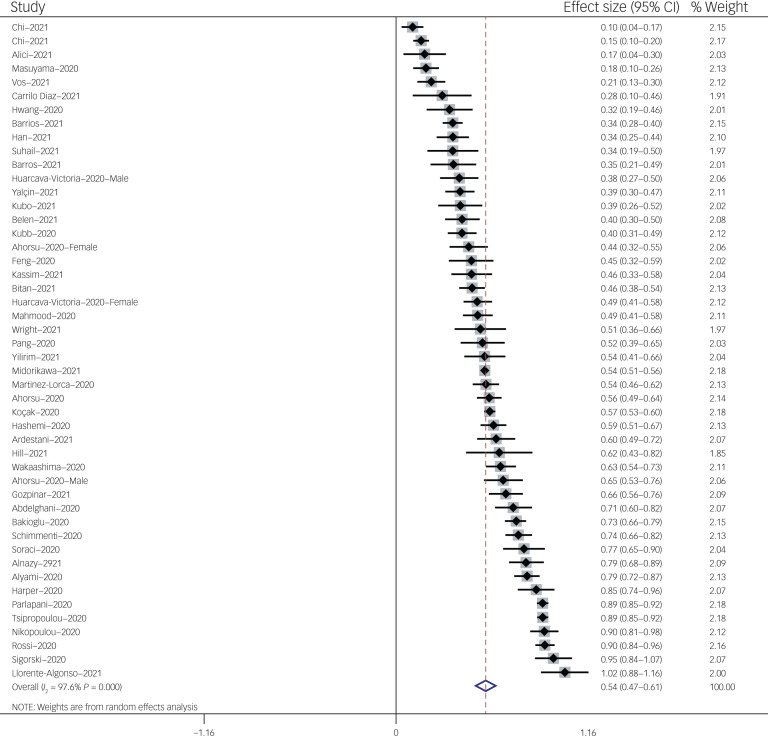
Forest plot displaying the estimated pooled Fisher's *z*-score in the association between fear of COVID-19 and anxiety.

**Fig. 11:**
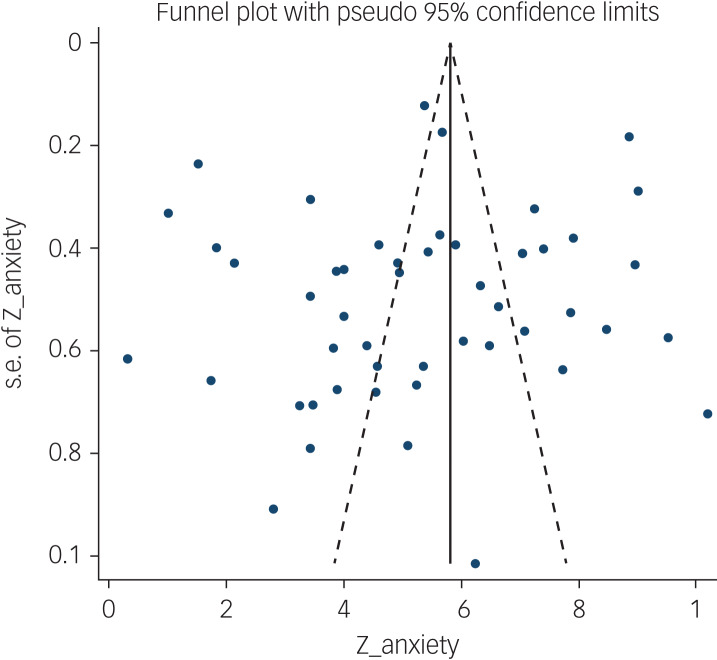
Funnel plot assessing publication bias in studies regarding the association between fear of COVID-19 and anxiety.

**Fig. 12:**
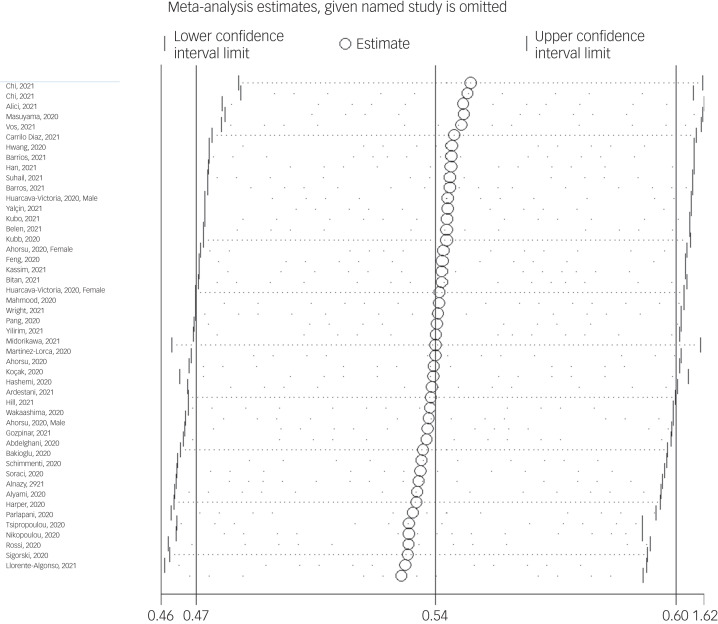
Sensitivity analysis plot assessing small study effect in the estimated pooled Fisher's *z*-score in the association between fear of COVID-19 and anxiety.

The association between fear of COVID-19 and anxiety was reported in 48 studies. The pooled estimated effect size showed strong correlation between fear of COVID-19 and anxiety, with a Fisher's *z*-score of 0.54 (95% CI 0.48–0.61, *I*^2^ = 97.6%, *τ*^2^ = 0.06). The forest plots are shown in Fig. 10. The probability of publication bias was assessed by Begg's test and funnel plot. Publication bias was not found in the association of fear of COVID-19 and anxiety based on Begg's test (*P* = 0.66) or funnel plot (Fig. 11). Sensitivity analysis showed that the pooled effect size was not affected by the single-study effect (*P* = 0.25; Fig. 12).

Subgroup analysis showed that association between fear of COVID-19 and anxiety was positive and higher, but not significant, among healthcare professionals compared with the general population (0.67 *v*. 0.53), and during the lockdown period compared with not being lockdown (0.70 *v*. 0.53). Meta-regression showed that age was the only significant moderator in the association of COVID-19-related fear and anxiety, explaining 15.5% variance in this association. Other variables did not influence heterogeneity or estimated pooled Fisher's *z*-score. Results of the subgroup analysis and meta-regression are shown in Tables 4 and 5.

#### Association between fear of COVID-19 and stress

**Fig. 13:**
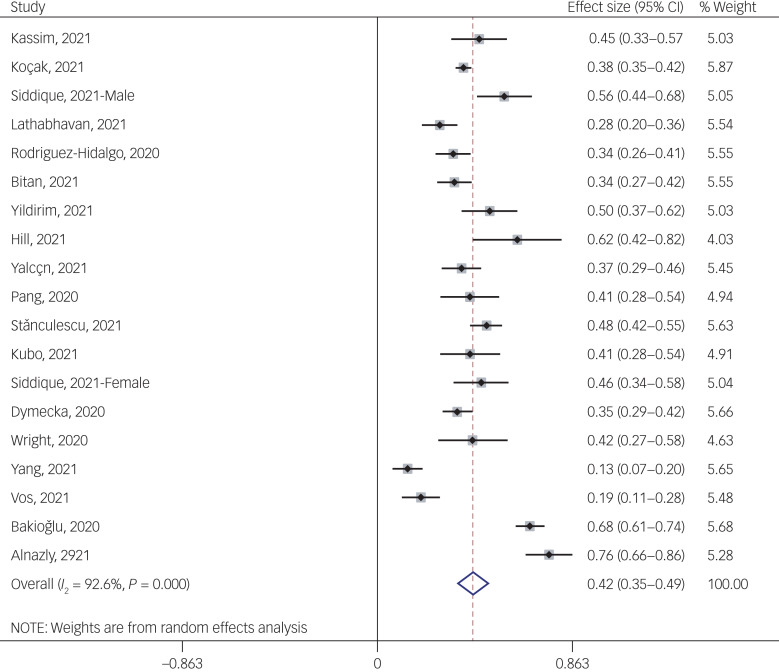
Forest plot displaying the estimated pooled Fisher's *z*-score in the association between fear of COVID-19 and stress.

**Fig. 14:**
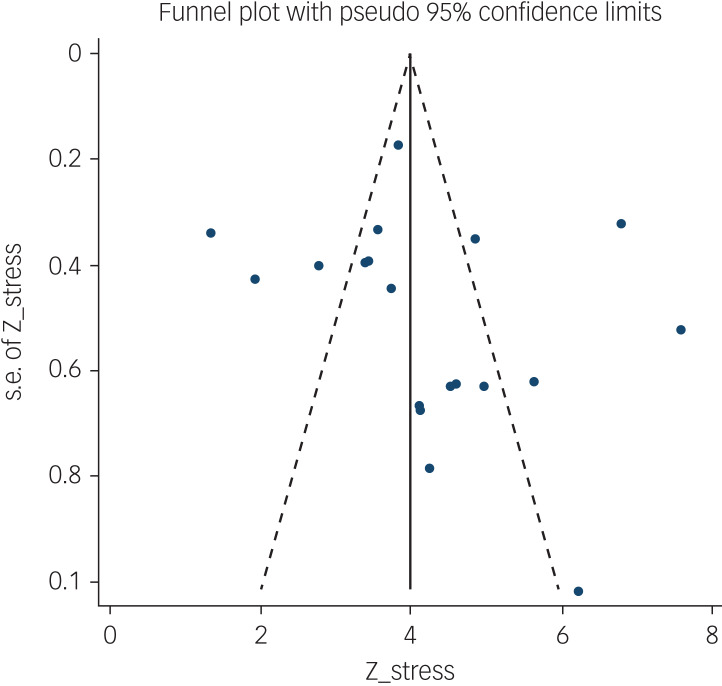
Funnel plot displaying the estimated pooled Fisher's *z*-score in the association between fear of COVID-19 and stress.

**Fig. 15:**
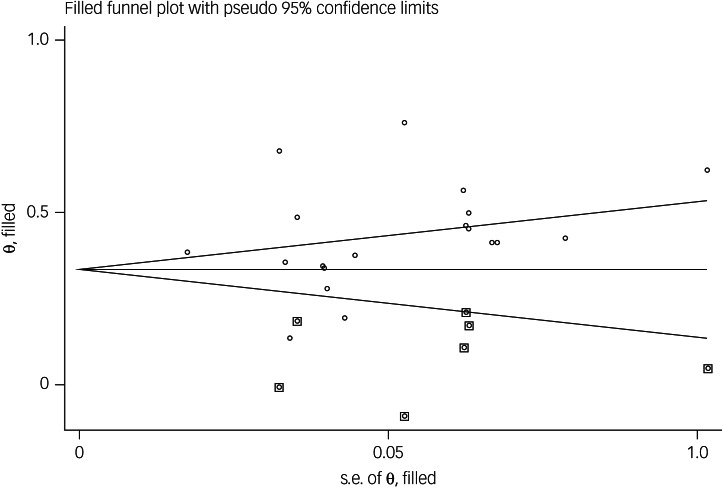
Corrected funnel plot assessing publication bias in the association between fear of COVID-19 and stress.

**Fig. 16:**
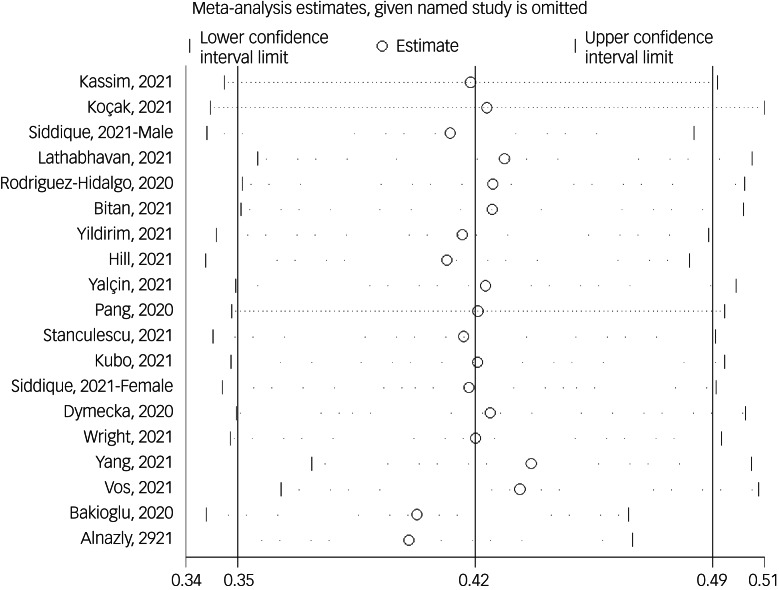
Sensitivity analysis plot assessing small study effect in the estimated pooled Fisher's *z*-score in the association between fear of COVID-19 and stress.

The association between fear of COVID-19 and stress was reported in 19 studies. The pooled estimated effect size showed moderate to strong association between fear of COVID-19 and stress, with a Fisher's *z*-score of 0.42 (95% CI 0.35–0.50, *I*^2^ = 92.6%, *τ*^2^ = 0.02). The forest plots are shown in Fig. 13. The probability of publication bias was assessed by Begg's test and funnel plot. Publication bias was not found in the association of fear of COVID-19 and stress based on Begg's test (*P* = 0.35), but was found in the funnel plot (Fig. 14). The fill-and-trim method was used to correct the results. In this method, seven studies were imputed, and the corrected results based on this method showed that pooled effect size of Fisher's *z*-score for association between fear of COVID-19 and stress was 0.34 (95% CI 0.26–0.41, *P* < 0.001). The funnel plot after trimming is shown in Fig. 15. Sensitivity analysis showed that the pooled effect size was not affected by the single-study effect (*P* = 0.42; Fig. 16).

Subgroup analysis showed that lowest heterogeneity was observed in studies conducted during lockdown period (35.2%). Although it appears that association between fear of COVID-19 and stress was positive and higher in studies with female-only participants (0.62 *v*. 0.42 in studies that included both genders) and studies that used FCV-19S to measure fear of COVID-19 (0.47 *v*. 0.27 in studies that used other scales), it was not significant. Subgroup analysis showed that association between fear of COVID-19 and stress was significantly higher among healthcare professionals compared with the general population (0.76 *v*. 0.41). Meta-regression showed that age and methodological quality of studies were the significant moderators in the association of COVID-19-related fear and stress, explaining 11.8% and 15.51 variance, respectively, in this association. Other variables did not influence heterogeneity or estimated pooled Fisher's *z*-score. Results of the subgroup analysis and meta-regression are shown in Tables 4 and 5.

#### Association between fear of COVID-19 and sleep problems

**Fig. 17:**
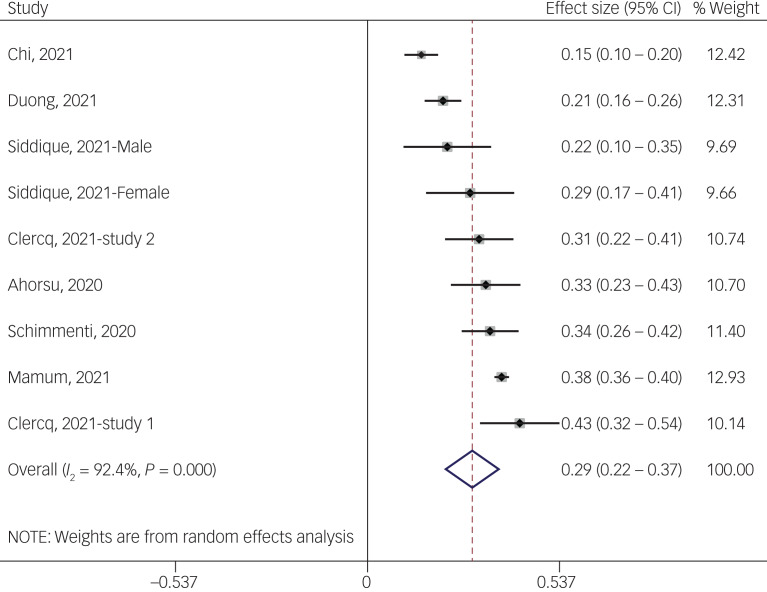
Forest plot displaying the estimated pooled Fisher's *z*-score in the association fear of COVID-19 and sleep problems.

**Fig. 18:**
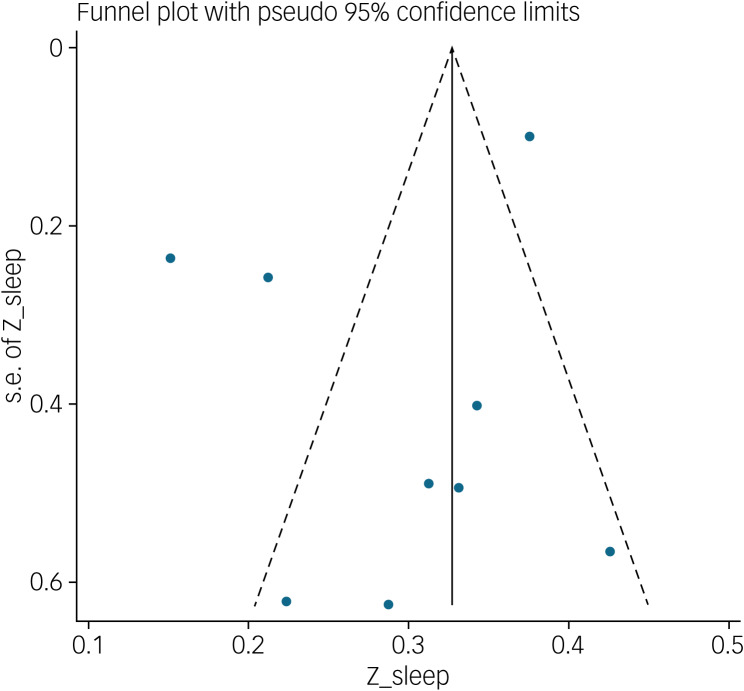
Funnel plot displaying the estimated pooled Fisher's *z*-score in the association between fear of COVID-19 and sleep problems.

**Fig. 19:**
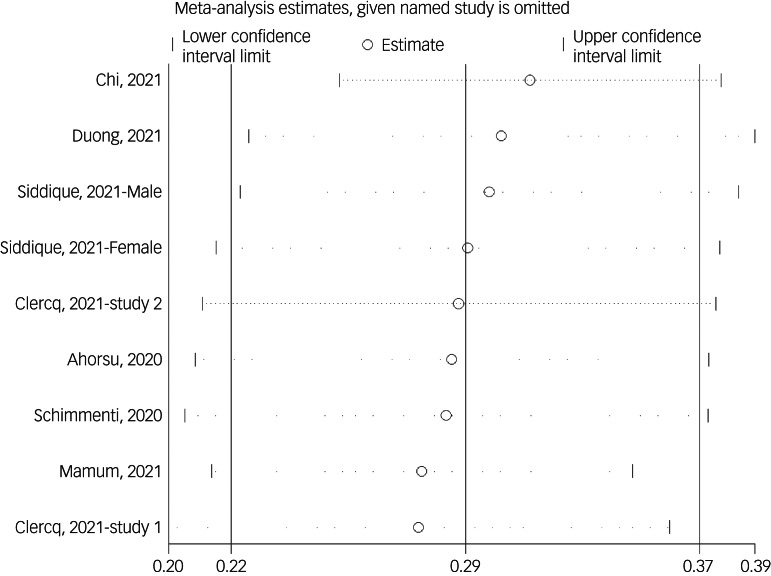
Sensitivity analysis plot assessing small study effect in the estimated pooled Fisher's *z*-score in the association between fear of COVID-19 and sleep problems.

The association between fear of COVID-19 and sleep problems was reported in nine studies. The pooled estimated effect size showed weak to moderate association between fear of COVID-19 and sleep problems, with Fisher's *z*-score of 0.29 (95% CI 0.22–0.37, *I*^2^ = 92.4%, *τ*^2^ = 0.01). The forest plots are shown in Fig. 17. The probability of publication bias was assessed by Begg's test and funnel plot. Publication bias was not found in the association of fear of COVID-19 and sleep problems based on Begg's test (*P* = 0.30) or funnel plot (Fig. 18). Sensitivity analysis showed that the pooled effect size was not affected by the single-study effect (*P* = 0.30; Fig. 19).

Meta-regression showed that age was the only significant moderator in the positive association of COVID-19-related fear and sleep problems, explaining 63.58% variance in this association. Other variables did not influence heterogeneity or estimated pooled Fisher's *z*-score. Results of the subgroup analysis and meta-regression are shown in Tables 4 and 5.

#### Association between fear of COVID-19 and mental health-related factors

**Fig. 20:**
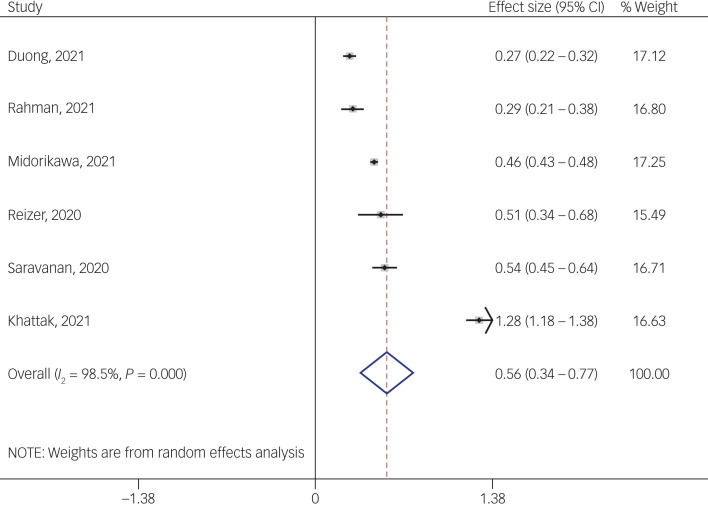
Forest plot displaying the estimated pooled Fisher's *z*-score in the association fear of COVID-19 and mental health-related factors. Arrow indicates that the CI does not fit the range of the x-axis.

**Fig. 21:**
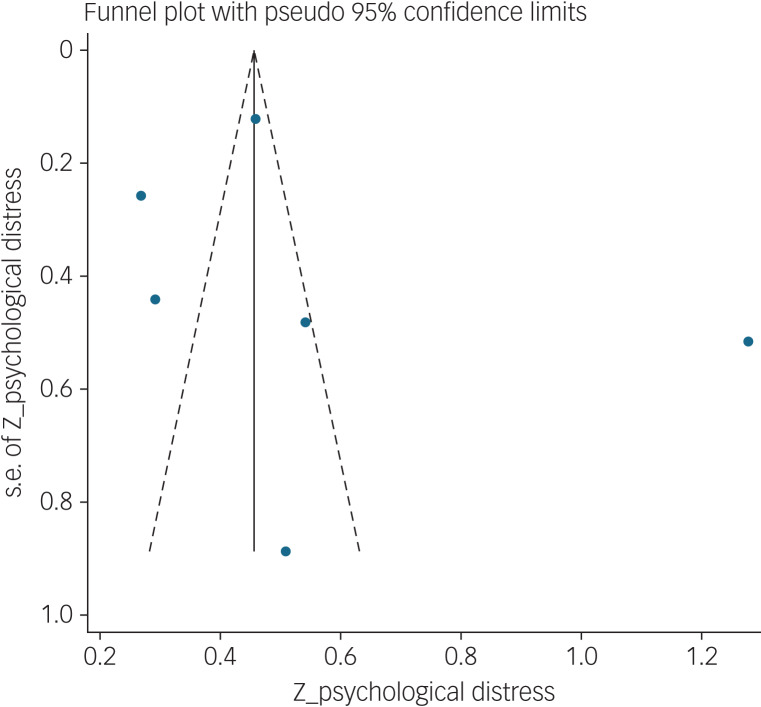
Funnel plot displaying the estimated pooled Fisher's *z*-score in the association between fear of COVID-19 and mental health-related factors.

**Fig. 22:**
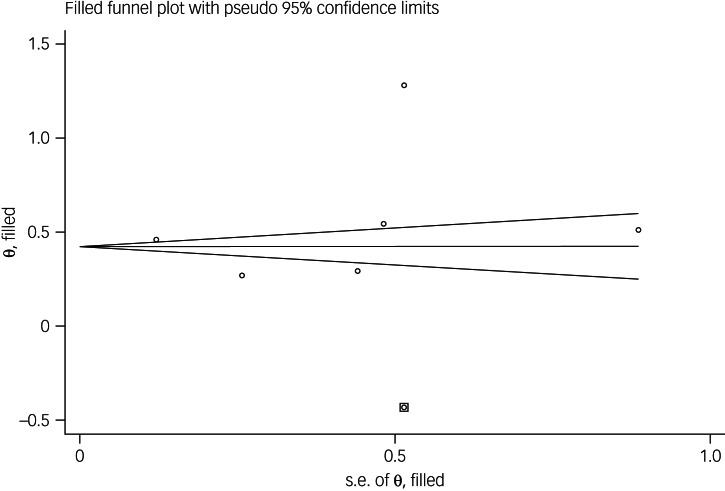
Corrected funnel plot assessing publication bias in the association between fear of COVID-19 and mental health-related factors.

**Fig. 23:**
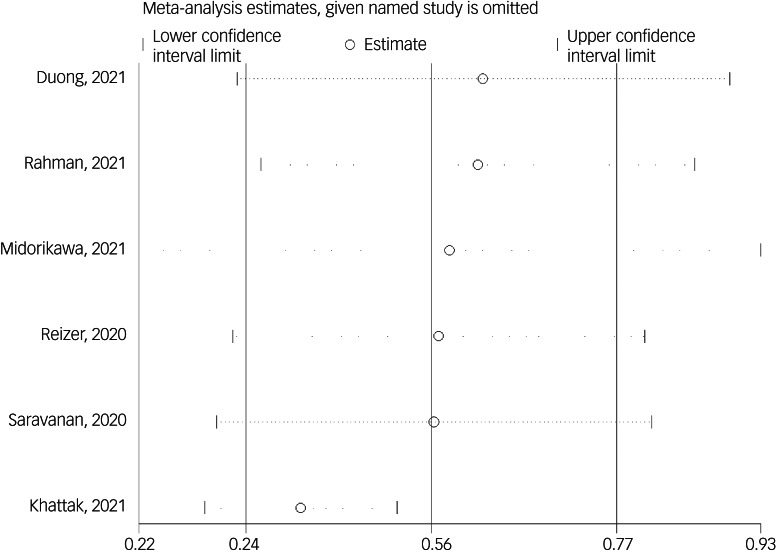
Sensitivity analysis plot assessing small study effect in the estimated pooled Fisher's *z*-score in the association between fear of COVID-19 and mental health-related factors.

The association between fear of COVID-19 and mental health-related factors was reported in six studies. The pooled estimated effect size showed strong association between fear of COVID-19 and mental health-related factors, with a Fisher's *z*-score of 0.56 (95% CI 0.34–0.77, *I*^2^ = 98.5%, *τ*^2^ = 0.07). The forest plots are shown in Fig. 20. The probability of publication bias was assessed by Begg's test and funnel plot. Publication bias was not found in the association of fear of COVID-19 and mental health-related factors based on Begg's test (*P* = 0.26), whereas the funnel plot appeared to be asymmetric (Fig. 21). The fill-and-trim method was used to correct the results. In this method, one study was imputed and the corrected results based on this method showed that pooled effect size of Fisher's *z*-score for the association between fear of COVID-19 and mental health-related factors was 0.42 (95% CI 0.16–0.67, *P* < 0.001). The funnel plot after trimming is shown in Fig. 22. Sensitivity analysis showed that the pooled effect size was not affected by the single-study effect (*P* = 0.58; Fig. 23).

Subgroup analysis showed that association between fear of COVID-19 and mental health-related factors was significantly higher among healthcare professionals (1 *v*. 0.41 for the general population). Such associations were also higher among studies that used FCV-19S to assess fear of COVID-19 (0.62 *v*. 0.27 in studies using other scales). Meta-regression showed that methodological quality score and instrument used to assess mental health-related factors explained 19.59% and 89.68% variance in this positive association. Other variables did not influence heterogeneity or estimated pooled Fisher's *z*-score. Results of the subgroup analysis and meta-regression are shown in Tables 4 and 5.

#### Association between fear of COVID-19 and mental well-being

**Fig. 24:**
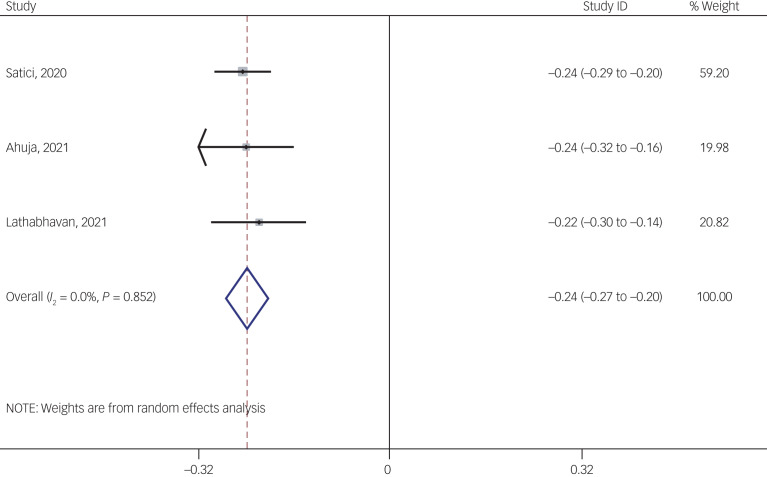
Forest plot displaying the estimated pooled Fisher's *z*-score in the association fear of COVID-19 and mental well-being.

**Fig. 25:**
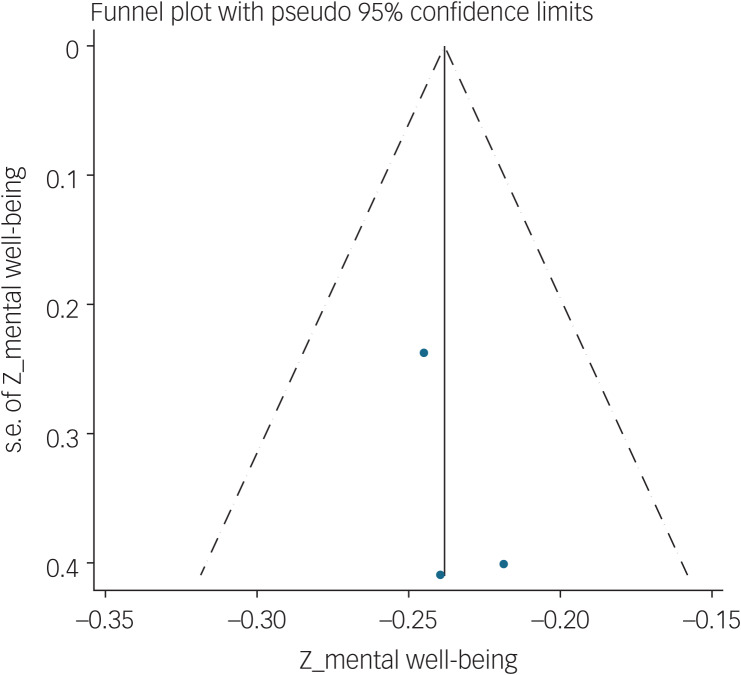
Funnel plot displaying the estimated pooled Fisher's *z*-score in the association between fear of COVID-19 and mental well-being.

**Fig. 26:**
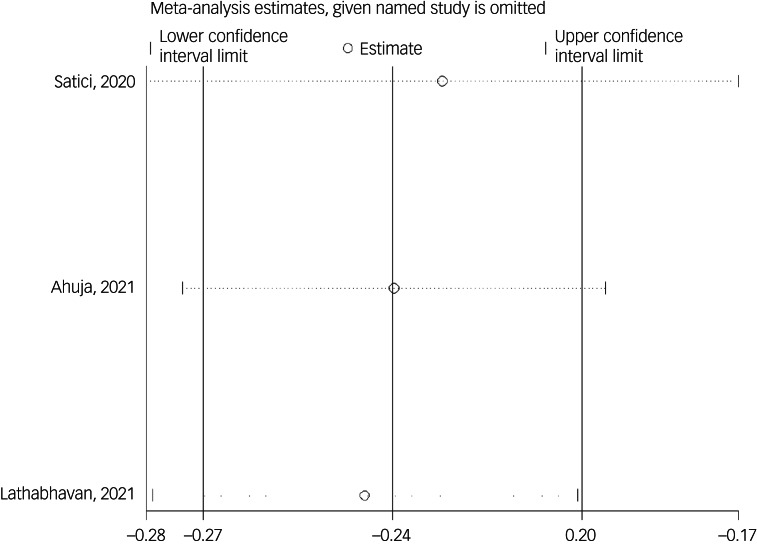
Sensitivity analysis plot assessing small study effect in the estimated pooled Fisher's *z*-score in the association between fear of COVID-19 and mental well-being.

The association of fear of COVID-19 with mental well-being was reported in three studies. The pooled estimated effect size showed negative and weak to moderate association between fear of COVID-19 and mental well-being, with a Fisher's *z*-score of −0.24 [95% CI −0.27 to −0.20, *I*^2^ = 0, *τ*^2^ = 0). The forest plots are shown in Fig. 24. The probability of publication bias was not found in the funnel plot (Fig. 25). Sensitivity analysis showed that pooled effect size was not affected by the single-study effect (*P* = 0.47; Fig. 26). Variables did not influence heterogeneity or estimated pooled Fisher's *z*-score. Results of the subgroup analysis and meta-regression are shown in Tables 4 and 5. Moreover, Table 6 summarises the pooled effect sizes for each studied variable associated with fear of COVID-19.

**Table 6:** Pooled effect sizes for studied factors correlated with fear of COVID-19

	Fisher's *z*-score	95% CI	*I*^2^	*τ*^2^
Depression	0.40	0.35–0.44	95%	0.02
Anxiety	0.54	0.48–0.61	97.6%	0.06
Stress	0.42	0.35–0.50	92.6%	0.02
Sleep problems	0.29	0.22–0.37	92.4%	0.01
Mental health-related factors	0.56	0.34–0.77	98.5%	0.07
Mental well-being	−0.24	−0.27 to −0.20	0.0%	0.00

## Discussion

To the best of our knowledge, the present systematic review and meta-analysis is the first to analyse the associations between fear of COVID-19 and a variety of mental health-related factors. More specifically, the systematic review and meta-analysis synthesised the evidence on the associations between fear of COVID-19 and depression, anxiety, stress, sleep problems, mental health-related factors and mental well-being during the COVID-19 pandemic period. After rigorous literature search, full texts of 298 papers were reviewed and 91 studies were included in the meta-analysis. Among the 91 studies, data from 88 320 participants in 36 countries were analysed. Moreover, the present meta-analysis showed that the mean estimation of fear of COVID-19 (using the FCV-19S) was 13.11, which indicates low levels of fear. More specifically, the score range of the fear was between 7 and 35, with a score of <21 indicating a low level of fear. Moreover, no significant gender differences were found in the fear of COVID-19.

The association between fear of COVID-19 and depression was moderate to strong (Fisher's *z* = 0.40), and a stronger association was observed among healthcare professionals (0.68) compared with the general population (0.37). The association between fear of COVID-19 and anxiety was strong (Fisher's *z* = 0.54), and no significant difference in the magnitude of association was found between healthcare professionals (0.67) and the general population (0.53). The association between fear of COVID-19 and stress was moderate to strong (Fisher's *z* = 0.42), and a stronger association was observed among healthcare professionals (0.76) compared with the general population (0.41). The association between fear of COVID-19 and sleep problems was weak to moderate (Fisher's *z* = 0.29). The association between fear of COVID-19 and mental health-related factors was strong (Fisher's *z* = 0.56), and a stronger association was observed among healthcare professionals (1 *v*. 0.41 for the general population) The association between fear of COVID-19 and mental well-being was weak to moderate (Fisher's *z* = −0.27). Meta-regression further showed that country, age, study quality, gender and measures for mental health-related factors were mostly non-significant moderators. Significant moderated effects were identified for age in anxiety and instruments on mental health-related factors (Table 5).

According to the meta-analysis results, fear of COVID-19 appears to contribute to mental health problems across different types, including depression, anxiety, stress, sleep problems, mental health-related factors and impaired mental well-being. However, the present findings were based on cross-sectional designs, which can only provide evidence of association rather than causality. Nevertheless, prior evidence and theories have supported that fear is a trigger for different types of mental health problems.^136–138^ Therefore, it can be tentatively concluded that fear of COVID-19 may lead to mental health-related problems based on the moderate associations found in the present meta-analysis. Furthermore, the associations found between fear of COVID-19 and other mental health-related factors appeared to be higher among healthcare professionals than individuals in the general population. This can be explained by the high levels of risk that healthcare professionals have been exposed to during the COVID-19 pandemic. More specifically, the workplaces of healthcare professionals are usually hospitals, and their jobs do not allow them to work from home. Therefore, they are likely to be exposed to environments with a much higher risk of COVID-19 infection than the work environments of the general population.^33,139^ Moreover, healthcare professionals usually have irregular work schedules, which may contribute to their mental health problems.^140–142^ Therefore, the association between fear of COVID-19 and mental health problems may be elevated when healthcare professionals are vulnerable in their mental health.

The instruments used for assessing fear of COVID-19 and other mental health-related factors are reported in Table 1. Diverse and inconsistent psychometric instruments were used for mental health-related factors in these studies. However, most of the studies used the FCV-19S to assess fear of COVID-19. The FCV-19S is a promising and robust instrument that has strong psychometric properties.^143,144^ Moreover, the FCV-19S^45^ contains only seven items, which is more practical to use in a busy setting, and provides accurate estimates of fear of COVID-19 in a short time (<5 mins). The FCV-19S has been validated in over 20 different languages.^143,144^ Therefore, it appears to be the most appropriate instrument assessing fear of COVID-19 for almost all of the studies reviewed in the present systematic review and meta-analysis. Future studies are recommended to use the FCV-19S if they want to assess the phenomenon of fear of COVID-19.

According to the findings derived from the present systematic review and meta-analysis, there are a number of implications. First, programmes to reduce fear of COVID-19, especially for healthcare professionals, are recommended during the pandemic period. More specifically, programmes with the support of strong theory (e.g. cognitive–behavioural therapy and meditation^145,146^) can be designed to tackle fear of COVID-19, and these may subsequently help maintain good mental health among both healthcare professionals and the general population during COVID-19 pandemic. Second, the associations between fear of COVID-19 and other mental health-related factors found in the present systematic review and meta-analysis indicate the importance of addressing the fear of COVID-19 together with other mental health-related factors. This may increase the effects of mental health improvement programmes during the pandemic. However, it should be noted that the present systematic review and meta-analysis found a large *I*^2^-value, which indicates the high levels of heterogeneity among the studies evaluated. However, large heterogeneity observed in the present findings is understandable because various factors that can increase the fear of COVID-19 together with the wide range of populations and measures were included in the meta-analysis.

### Strengths and limitations

There are some strengths in the present systematic review and meta-analysis. First, the mean estimation of fear of COVID-19 and its associations with other mental health-related factors were estimated across different countries worldwide. Therefore, the analysis provides a contextualised picture regarding the psychological phenomenon during the COVID-19 pandemic. Second, the methodology of the present systematic review and meta-analysis was rigorous, given that each analysed study had been evaluated for their methodological quality by the NOS checklist. Moreover, a thorough literature review was conducted utilising five academic databases. In addition to the main and secondary outcomes, the synthesised findings were checked for their stability by additional analyses, including subgroup analysis and meta-regression. Third, the present findings have relatively high generalisability because the analysed data come from a large sample size (*N* = 88 320) across 36 countries.

There are also some limitations in the present systematic review and meta-analysis. First, fear of COVID-19 and other mental health-related factors analysed in the present meta-analysis were assessed by different psychometric instruments across the studies (e.g. Depression, Anxiety and Stress Scale-21 and Hospital Anxiety and Depression Scale). Therefore, the different item descriptions and scoring method used in these measures may cause biases in estimation. However, meta-regression in the present systematic review and meta-analysis shows that almost all of the measures had no significant effects on the synthesised results. Therefore, this limitation may not be serious. Second, all studies, except for one, that were analysed in the present systematic review and meta-analysis employed a cross-sectional design. Without the time factor in the study design, the associations found in the present findings do not have strong causal evidence in relation to the variables under investigation. Therefore, future studies using longitudinal designs are warranted to provide additional evidence in more rigorously exploring the causal relationships between fear of COVID-19 and other mental health-related factors. Third, although the present systematic review and meta-analysis analysed 91 studies, only three of them^46,118,126^ assessed the associations between fear of COVID-19 and mental well-being. Therefore, further studies are needed to corroborate the evidence regarding the association between fear of COVID-19 and mental well-being.

In conclusion, the present study found that the fear of COVID-19 had associations with a variety of mental health-related factors, from slightly weak to relatively strong magnitudes. Moreover, healthcare professionals, as compared with the general population, had stronger magnitudes in the associations between fear of COVID-19 and some mental health-related factors (including depression, stress and mental health-related factors). Therefore, programmes on reducing fear of COVID-19 and improving mental health for both healthcare professionals and the general population are warranted during the ongoing pandemic.

## Data Availability

The authors confirm that the data supporting the findings of this study are available within the article.
